# Training and Transfer of Cue Updating in Older Adults Is Limited: Evidence From Behavioral and Neuronal Data

**DOI:** 10.3389/fnhum.2020.565927

**Published:** 2020-12-04

**Authors:** Jutta Kray, Nicola K. Ferdinand, Katharina Stenger

**Affiliations:** ^1^Department of Psychology, Saarland University, Saarbrücken, Germany; ^2^Department of Psychology, Bergische Universität Wuppertal, Wuppertal, Germany

**Keywords:** task-switching training, cue updating, Cue-P3, older adults, transfer

## Abstract

Cognitive control processes, such as updating task-relevant information while switching between multiple tasks, are substantially impaired in older adults. However, it has also been shown that these cognitive control processes can be improved by training interventions, e.g., by training in task switching. Here, we applied an event-related potential (ERP) approach to identify whether a cognitive training improves task-preparatory processes such as updating of relevant task goals. To do so, we applied a pretest-training-posttest design with eight training sessions. Two groups of older adults were either trained in task switching (treatment group) or in performing single tasks (control group) and we compared their performance to a group of untrained younger adults. To foster cue updating in the treatment group, we applied a cue-based switching task in which the two task cues were randomly selected prior to target presentation so that participants had time to prepare for the upcoming task. In contrast, the control group also received task cues but those were redundant as only one task had to be performed. We also examined whether training in cue updating during task switching can be transferred to a similar cognitive control task measuring updating of context information, namely a modified version of the AX-Continuous Performance Task (AX-CPT). The results revealed training-specific improvements in task switching, that is, a larger improvement in blocks requiring switching in comparison to single tasks at the behavioral level. In addition, training specific-effects were also found at the neuronal level. Older adults trained in cue updating while switching showed a reduction in mixing costs in the cue-related P3, indicating an improvement in preparatory updating processes. Additionally, P3 topography changed with training from a very broad to a parietally focused scalp distribution similar to the one found in younger adults. However, we did not obtain training-specific improvements in context updating in the AX-CPT neither at the behavioral level nor at the neuronal level. Results are discussed in the context of the ongoing debate on whether transfer of cognitive training improvements is possible.

## Introduction

It is a well-documented finding in cognitive aging research that a variety of cognitive domains, such as working memory, inhibition, and cognitive flexibility show a substantial decline with increasing age (for reviews, Nyberg et al., 2012; Hartshorne and Germine, 2015). This is of importance in light of a growing elderly population and a prolonged life expectancy. Hence, a key challenge for researchers in the field of cognitive aging is (a) to identify conditions under which cognitive decline may be reversed or cognitive functioning can at least be maintained, (b) to investigate which training interventions not only lead to performance improvements in the trained tasks but also to better performance in untrained tasks (Binder et al., 2015), and (c) how these processes are reflected in the brain (e.g., Brehmer et al., 2014; Dörrenbächer et al., 2020).

In the meantime, there is not only evidence that the ability to improve the level of cognitive functioning is preserved even in very old age (Lövdén et al., 2010; Kühn and Lindenberger, 2016). There is also evidence that training interventions aiming at improving cognitive control functioning in older adults lead to performance gains in untrained cognitive tasks (for a review, Kray and Dörrenbächer, 2019; for a meta-analysis, see Karbach and Verhaeghen, 2014). Cognitive control is required in situations in which we need to adapt our thoughts and actions according to internal task goals in the context of changing environments. A common component of cognitive control in many cognitive tasks is representing and maintaining task-relevant knowledge (of the actual task or context) in working memory (Braver and Barch, 2002; Braver, 2012). Moreover, as more specific components of cognitive control, Miyake and Friedman (2012) identified the updating of task-relevant information and the switching between task rules.

The primary goal of the present study was to train older adults in the implementation of cognitive control by means of a task-switching training. In particular, because it is known that with aging the ability to apply proactive, i.e., preparatory, control processes declines rapidly and leads to a shift toward relying on reactive control (cf. West and Schwarb, 2006; Paxton et al., 2008; Braver, 2012), we aimed at investigating whether an intensive task-switching training would improve early task-preparatory processes, such as updating task-relevant goals in advance, which may also result in better switching performance. Therefore, we applied EEG measurement that allows to assess fine-grained temporal processes during preparing the upcoming task.

### Age Differences in Cognitive Control

To date there is a variety of empirical evidence for age differences in cognitive control as measured with the task-switching paradigm (for a review, see Kray and Ferdinand, 2014; for a meta-analysis, see Wasylyshyn et al., 2011). In this paradigm, participants are instructed to perform two (or more) different cognitive tasks A and B, for instance, to categorize pictures as belonging to the category of fruits or of vegetable in task A, and to categorize the size of the pictures as small or large in task B. Participants perform these tasks in two types of blocks, the so-called single-task blocks and mixed-task blocks. In single-task blocks, they only perform one task A or B in isolation, while in mixed-task blocks, they are instructed to switch between both tasks A and B either in a predictable or in a random order (for overviews, see Kiesel et al., 2010; Grange and Houghton, 2014). Two types of costs can be derived from this paradigm: (1) Mixing costs (also termed global or general shifting costs) that are defined as difference in performance between mixed-task and single-task blocks. Mixing costs are considered to reflect the ability to deal with the switching situation, that is, to maintain and select between two task-sets, such as in mixed-task blocks, as compared to maintain only one task in single-task blocks. (2) Switching costs (also termed local or specific shifting costs) are defined as the difference in performance between switch and repeat trials *within* mixed-task blocks. These costs are assumed to reflect the ability to disengage from a previous task rule and to shift to the other task rule.

From behavioral studies, there is quite some evidence that older adults show larger mixing costs than younger adults, suggesting age-related impairments in maintaining multiple task sets. Moreover, age differences in switching costs are substantially smaller than in mixing costs (for reviews, Kray and Ferdinand, 2014; Gajewski et al., 2018). Further empirical support for the differential age-related changes in these types of task-switching costs comes from a meta-analytic study on age differences in task switching (Wasylyshyn et al., 2011). These findings suggest that older adults' deficits are due to being in a switch situation and requiring the selection between tasks and their maintenance and not by executing the task switch itself. However, it has been shown that age differences in mixing costs can be reduced by increasing the time to prepare for the next task (Kramer et al., 1999; Kray and Lindenberger, 2000; Kray, 2006; Whitson et al., 2012), or by instructing participants to verbalize the next task prior to target presentation, that is, when using verbal cues that facilitate task preparation in the absence of external task cues (Kray et al., 2008). In contrast, the pattern of much larger mixing costs relative to switching costs has primarily been observed in cued task-switching paradigms in which the two tasks are randomly presented and participants need to update which task to prepare depending on an external cue (Kray, 2006). One explanation for this pattern of findings is that older adults, in contrast to younger adults, tend to update the task also in repeat trials (when it is not necessary) and not only in switch trials, therefore switch costs are low or sometimes even negative in comparison to mixing costs (Karayanidis et al., 2011; Whitson et al., 2012). This updating deficit is particularly found in situations of uncertainty, when the targets are ambiguous as they contain features associated with both relevant tasks (Mayr, 2001). Alternatively, it has been suggested that older adults prefer a reactive control mode during performing a task, meaning that they are less engaged in advance preparation according to cues presented and update the task (or cue information) only when the target is presented. If they do so in repeat and switch trials, responses are much slower in mixed as compared to single trials while difference between repeat and switch trials are relatively low. In contrast, young adults prefer an active control mode in which they prefer and update the task representation in advance and if needed (Braver and Barch, 2002). On the basis of these theoretical consideration and previous empirical findings, we will focus on the comparison between single blocks and mixed blocks in the present study.

Because of their high temporal resolution, neurophysiological measures like event-related potentials (ERPs) can further contribute to determine the sources of age differences in mixing costs. In particular, ERPs allow to separate proactive processes required for task preparation—which are supposedly difficult for older adults—from those required for later task execution and response selection processes including reactive control processes (for reviews, Karayanidis and Jamadar, 2014; Gajewski et al., 2018). To investigate whether proactive control in older adults can be improved by a specific switching training, we compared two older training groups, a task-switching group that practiced to continuously update task-cue information, and therefore only performed mixed-task blocks in which the two tasks were randomly selected. In contrast, the single-task group only performed both tasks in separate blocks so that attending to the cue information was not necessary.

An ERP component that occurs during task preparation in cued task-switching paradigms and that is typically elicited by the presentation of a task cue is the cue-related P3. It is a parietal positivity that occurs about 400–600 ms after cue-onset (for a review, see Gajewski et al., 2018). Because it is usually more pronounced for switch as compared to non-switch trials (e.g., Kieffaber and Hetrick, 2005; Nicholson et al., 2005; Swainson et al., 2006; Lavric et al., 2008), it has also been labeled sustained posterior positivity (cf. Kopp et al., 2014) or (when measured as the difference wave between switch and repeat trials) switch positivity (Karayanidis et al., 2003, 2011). This parietal positivity has been linked to the idea of the classic P300/ P3b account (Donchin, 1981; Donchin and Coles, 1988; Polich, 2007), that has been assumed to reflect updating of task relevant knowledge, an idea that is corroborated by the finding that larger cue-P3s are related to smaller switch costs (e.g., Karayanidis et al., 2009, 2011; Elchlepp et al., 2012). However, it recently has been shown that the cue-related P3 and the target-related P3 are functionally distinct mechanisms, the one associated with the updating of higher-order rules (or task sets), and the other with updating of lower-level S-R rules (Barceló and Cooper, 2018a,b). Here we will focus on the updating of task rules instead of S-R rules. There is also evidence that cue-related P3 does not reflect a unitary updating process, but different subcomponents of the cue-P3 represent different aspects of updating. In particular, in cued switching paradigms an early and late positivity can be differentiated that are assumed to be associated with the intention to switch and updating the now relevant task set, respectively (cf. Karayanidis and Jamadar, 2014).

Older adults usually show less efficient task-set updating: When comparing mixed with single task blocks, older adults show a longer cue-P3 latency than younger adults (e.g., Kray et al., 2005; Eppinger et al., 2007; West and Travers, 2008). Additionally, when comparing switch and repeat trials, younger adults display larger cue-P3s on switch than repeat trials. In contrast, older adults show a reduced amplitude difference between these two trial types. This finding is interpreted as older adults needing to update task sets on each trial when they are in a switching context, no matter whether it is actually necessary or not (Eppinger et al., 2007; Friedman et al., 2008; Whitson et al., 2014). At the same time, ERPs show evidence for an additional recruitment of frontal brain regions in older adults, as can be inferred from a frontal shift in the topography of the cue-P3 (Kray et al., 2005; Eppinger et al., 2007; Karayanidis et al., 2011). This has been interpreted as a compensatory mechanism which helps older adults to keep their performance up (Friedman, 2008; Reuter-Lorenz and Cappell, 2008).

Another paradigm that can be used to measure preparatory cognitive control processes, such as updating task and response rules, is the AX-continuous performance task (AX-CPT). In this task, stimuli are presented in cue-target pairs and performance is compared across four types of cue-probe combinations. Participants are instructed to respond (e.g., with a right button press) to a specific target pair “AX” (when the target “X” is following the cue “A”) that is presented in 70% of the all trials to induce a strong response bias. In AY trials, another target (e.g., M, L, K) is following the cue “A,” while in BX trials the target “X” is preceded by other cues (e.g., P, T, S). Finally, in BY trials neither the cue A nor the target X is presented. Each of the three combinations (AY, BX, BY) is presented only in 10% of the trials and participants are instructed to respond with a left button press. Two types of control processes have been identified with this type of paradigm. If participants produce more AY than BX errors, they are strongly engaged in advance preparation of the response associated with the A cue (proactive control). In contrast, BX errors occur when participants are less engaged in advance preparation and press the wrong response button because they fail to correctly reactivate the preceding cue information (reactive control mode). In a number of studies, Braver and colleagues found that whereas younger adults showed strong engagement in task preparation processes, like updating and maintaining the cue (context) information (proactive control), older adults had deficits in advance preparation and instead needed to reactivate the cue information when confronted with the target (reactive control; Braver et al., 2005; Rush et al., 2006; Paxton et al., 2008; for a review, see Braver and Barch, 2002).

This general pattern has also been confirmed in ERP studies with a modified version of the AX-CPT, as the former paradigm is less suitable for ERP research given the distribution and low number of trials in critical conditions. In this version, two types of trials can be separated, context-dependent and independent trials. Similar to the cued task-switching paradigm, two cues (the context-dependent ones) induce the preparation of two response alternatives, but only one of them is actually selected after target presentation. In the other half of the cases, two different cues are redundant (context-independent) as the response selection is clearly associated with the target only so that preparation and cue updating is not needed. In younger adults, larger P3 amplitudes after cue presentation have been found in trials where the response is dependent on the preceding cue (context-dependent trials) than in trials where the response is independent on the cue (context-independent trials). This has been interpreted as younger adults ability to flexibly adapt to the more difficult conditions that need more preparatory updating of task-relevant information (Lenartowicz et al., 2010; Schmitt et al., 2014a,b). In contrast, older adults' P3 amplitudes did not differ between conditions, suggesting that older adults needed to update context information on every trial, even when it was not required (Schmitt et al., 2014a,b), similar to the results obtained in cued-task switching. Of importance for the present study, it has been found that these preparatory updating processes can be strengthened in the elderly, e.g., by extended practice and directed strategy training (Paxton et al., 2006; Braver et al., 2009) or by the prospect of a reward (Schmitt et al., 2015, 2017).

In sum, preparing an upcoming task depending on cues and the respective neuronal correlates have been investigated with cued task-switching paradigms in which a cue either indicated a task switch or task repetition, and in the modified AX-CPT in which cues either were informative for response selection or not. Hence, in both paradigms younger adults recruit more cognitive control (as indexed by larger cue-P3 amplitudes) when needed (after a cue switch or in context-dependent trials), while older adults also invest in control when it is not needed.

### Training of Task Switching

There is an ongoing debate in cognitive training research mainly about whether cognitive training gains can be transferred and generalized to untrained cognitive tasks (e.g., Novick et al., 2019). While a lot of studies have examined age differences in near and far transfer effects in the domain of working memory and multitasking (Anguera et al., 2013; Strobach et al., 2016), there is also some evidence on the effectiveness of task-switching training (for recent reviews, Karbach and Kray, 2016; Kray and Dörrenbächer, 2019). It has been shown that training switching by performing mixed-task blocks as compared with training only single tasks leads to a larger reduction of mixing and switching costs, and this reduction was even more pronounced in older than in younger adults (Karbach and Kray, 2009; Kray and Fehér, 2017). For both, younger and older adults, larger transfer effects, i.e., a larger reduction of mixing costs under a variable training in which participants received a new set of stimuli and tasks in each of the training sessions were found (cf. Karbach and Kray, 2009). In addition, older adults also showed larger transfer effects of task-switching training when the ambiguity and by this the interference between two tasks was high (Kray and Fehér, 2017). Given these previous findings, we decided to apply a variable training with ambiguous stimuli in the present study.

There are only a few studies examining different training conditions (e.g., cognitive, physical, relaxation) on changes in neuronal correlates of task switching in older adults. One study by Gajewski and Falkenstein (2012) found that the target P3 was enhanced after the cognitive training intervention compared to the control group and the physical and relaxation group but unspecifically on single, repeat and switch trials, which was interpreted as higher cognitive resources to perform the actual task. In a recent study, the effects of multi-domain cognitive training on task preparation and task execution was examined in a switching task in which participants were asked to switch between responding to the word measuring or colors of Stroop stimuli (Küper et al., 2017). Interestingly, the cognitive training intervention, as compared to the active and passive (social) control group, influenced processing in the cue processing stage. Here, they found that in the cognitive training group, the cue-locked P3 increased from pretest to posttest for repeat trials at all electrodes (Fz, Cz, and Pz), and in the active control group only at the central electrode while such an increase was not obtained in the passive control group. This finding was interpreted by the authors as better maintenance of task rules from one trial to the next under switching conditions. The effect was unspecific to the training intervention as it was also obtained in the active control group. However, the cognitive training was a multi-domain training including memory, speed, and reasoning tasks that may not be similar enough to induce transfer of training, in particular, a boost in the updating of task rules.

### The Present Study

We already have some evidence from previous training studies that training in task switching is useful and effective and can lead to performance improvements in untrained similar switching tasks and other cognitive tasks in older adults. The primary question of this study was whether potential training gains mainly result from improved task preparation and whether such improvements can be transferred to other cognitive control tasks. Therefore, we applied an ERP approach to determine changes in cue processing during the anticipation of the next task with a cued-based task-switching paradigm at pre- and post-test. To examine the effects of training on cue updating, older participants were assigned to two different training groups. One group received eight practice sessions of switching by performing only mixed-task blocks in which the cue was relevant to correctly perform the next task (task-switching group). The other group, as active control group, only received single-task blocks (single-task group) during the eight practice sessions in which the cue was non-informative for the task. Note that the training was variable, meaning, that all participants performed a new set of stimuli and tasks in each of the eight training sessions and all stimuli were ambiguous to induce interference between both tasks, as we found larger transfer effects under these training conditions in two previous studies (Karbach and Kray, 2009; Kray and Fehér, 2017). To determine transfer effects, our participants also performed a modified AX-CPT task that, similar to the cued task-switching paradigm, required the updating of cue-relevant information (see Methods section). Here, ERPs were also measured to the onset of the cue. We also assessed a group of younger adults at pre- and post-test in both tasks to determine age differences in cognitive control processes.

On the behavioral level, we expected that both training groups would improve during the training sessions, that is, we should find faster reaction times and less errors with increasing practice. We also expected transfer effects, that is, a larger reduction of task-switching costs and a larger reduction in the context effect in the task-switching training than in the single-task training group. On the neuronal level, we focused on the cue-P3 in the preparation interval and expected changes in cue-P3 amplitudes, indexing engagement in updating task rules. In particular, we expected updating to become more specific to the trials where it is needed (switch trials) and by this result in a decrease of mixing and an increase or emergence of switching costs. If practice effects in updating cue-relevant information can be transferred to another task, we would also expect changes in the cue-P3 of the AX-CPT. More specifically, a difference in P3 amplitudes between context-dependent and context-independent trials should emerge after the training resulting from a more flexible investment of cognitive control in those trials where it is actually needed.

## Methods

### Participants

Overall, 64 older adults (33 males) were willing to participate and complete the intensive training, while 31 younger adults (18 males) only participated in the pretest and the posttest sessions. Participants were recruited through a newspaper article and from a subject pool of Saarland University. All participants signed informed consent in advance and received a monetary compensation of €8 per hour for their attendance. The older adults additionally received a reimbursement of €20 for travel expenses. The study procedure and the written informed consent were approved by the local ethics committee at Saarland University.

According to self-reports, all participants were native German speakers, reported normal or corrected to normal vision and hearing, and none of the participants reported neurological or psychological disorders. Moreover, all participants were right-handed as measured with the Edinburgh Inventory (Oldfield, 1971). Characteristics of the final sample are shown in Table 1. In line with previous studies on aging and the two-component model of intelligence (Baltes et al., 1999), we found significant age differences in processing speed as measured with the Digit Symbol Substitution Test (DSST, adapted from Wechsler, 1981), that is, a typical slowing in perceptual speed of processing in older adults as compared to younger adults, *F*_(1,92)_ = 64.61, *p* < 0.001, $\eta_{\text{p}}^{2}$ = 0.41. In contrast, in a semantic knowledge test, the Spot-a-Word Test (adapted from Lehrl, 1977), older adults achieved a higher score than younger adults, *F*_(1,92)_ = 51.96, *p* < 0.001, $\eta_{\text{p}}^{2}$ = 0.36.

**Table 1:** Characteristics of the sample and baseline measurement of cognitive tasks at pretest.

	**Group**		
	**Younger adults (*n* = 31)**	**Single-task training (*n* = 34)**	**Task-switching training (*n* = 30) adults (*n* = 31)**
	***M (SD)***	***M (SD)***	***M (SD)***
Mean age (years)	22.9 (2.74)	70.35 (4.68)	68.27 (3.97)
DSST test score	61.97 (11.2)	42.29 (8.94)	46.30 (9.70)
Spot-a-word test score	22.29 (3.5)	27.68 (3.80)	27.43 (2.78)
RT mixing costs (ms)	67 (61.0)	123 (93.0)	124 (96.1)

*DSST, Digit Symbol Substitution Test; RT, Reaction Time*.

Importantly, in order to avoid baseline differences in cognitive measures between the two training groups, these two groups were matched according to their performance in the perceptual speed task and the magnitude of mixing costs at pretest. As can be seen in Table 1, the two trainings groups did not significantly differ in both measures (*p* = 0.09 and *p* = 0.98, respectively). Moreover, they also did not differ in mean age (*p* = 0.06).

### Study Design and Procedure

To measure training and transfer effects, we used a pretest-training-posttest design. The pretest sessions included the assessment of cognitive functioning by means of a cognitive test battery as well as the baseline measurement of neuronal correlates of cognitive control functioning by means of EEG recordings (of about 180 min) and functional imaging (fMRI) that was measured in a separate session (of about 150 min). The pretest EEG and fMRI sessions were identical to the posttest session. Here, we will report only the results from the EEG sessions (for the fMRI results, see Dörrenbächer et al., 2019, 2020). Only the older adults performed the eight training sessions between pre- and post-test that were spaced over 4 weeks. Thus, the participants received the training intervention twice a week for about 45 min. We tested participants individually in the training sessions by one experimenter and at pretest and posttest by two experimenters. Each of the sessions will be described in detail below.

#### Pre- and Post-test Sessions

In the pre- and post-test sessions, each participant performed three cognitive control tasks: a switching task, a context-updating task (modified AX-CPT), and a working-memory filtering task, while the EEG was recorded. As task-preparatory processes cannot be observed in the memory-filtering task, we will focus on the results of the switching task and the context-updating task. The experimental tasks for the pre- and post-test as well as for the training sessions were programmed using E-Prime® 2.0 Professional (Psychology Software Tools, Inc, 2012).

##### Measurement of Task Switching

To measure task-switching performance, we applied a modified version of the task-switching paradigm as used in the training study by Karbach and Kray (2009). In this paradigm, participants are instructed to perform two categorization tasks, either in isolation (i.e., single-task blocks), or they have to switch between them (i.e., mixed-task blocks). Targets were pictures that had to be categorized according to semantic meaning either as fruit or vegetable (task A), or according to a perceptual feature as small or large in size (task B) by pressing one of two buttons on a response pad. Hence, the stimulus-response mappings of both tasks were overlapping because one feature of each of the two tasks was mapped onto the same response key. Participants were instructed to use their left and right index fingers for responding as well as to respond as quickly and accurately as possible. In contrast to the previous study, participants received cues indicating the next task A or B that were presented in a random order. Cues consisted of two letters indicating the food task (ES = “Essensaufgabe”) or the format task (FO = “Formataufgabe”) in advance of the target stimulus.

Target stimuli were 32 colored pictures of food items (16 fruits, 16 vegetables) adapted from the Snodgrass and Vanderwarts' pictorial set (Rossion and Pourtois, 2004). All targets were presented in a pseudorandom order in either small size (90 × 90 pixels) or large size (220 × 220 pixels) at the center of the computer screen.

In the practice phase, participants first performed two single-task blocks of 12 trials and two mixed-task blocks of 12 trials. Thereafter, they performed eight experimental blocks (four single-task blocks and four mixed-task blocks). Each block consisted of 41 trials while the first trial as a re-start trial was always excluded from data analyses. The mixed-task blocks consisted of 20 repeat and 20 switch trials presented in a random sequence. Each block consisted of an equal number of stimulus and response types. Performance feedback of mean reaction times and error rates was given at the end of each block. Stimulus-response assignments were counterbalanced across participants as well as the order of single-task blocks. Testing time lasted about 25 min.

The trial procedure was identical for single and mixed trials. Each trial started with a 300 ms fixation cross, followed by the cue (i.e., ES or FO), which was presented for 800 ms. After the cue, a second fixation cross was presented for 1,000 ms, followed by the target that was displayed for 1,800 ms. Responses had to be executed within this given time window. Otherwise, the trial was excluded from further analyses. The inter-trial interval (ITI) lasted for 500 ms.

##### Measurement of Context Updating

To measure context updating, we applied a modified AX-CPT (Lenartowicz et al., 2010), that was further adapted to examine age differences in neuronal correlates of context updating in studies from our lab (e.g., Schmitt et al., 2014a).

In this version of the AX-CPT, participants were instructed to respond to four different cue-target combinations by pressing either the left or right response button (i.e., if-then rules). The task consisted of two trial types. In context-dependent trials (c-dep), correct responses to targets were depended on the preceding cue. For instance, if the female person was followed by a bird, then participants should press the left key, and if the male person was followed by a cat, then should press the right key. In context-independent (c-indep) trials, responses to the targets did not depend on the preceding cue. Nevertheless, the same type of instructions was given. For instance, if the female person was followed by a fish, then participants should press the left key, and if the male person was followed by the fish, then participants should press the left key. In context-independent trials, the cue can be neglected in order to select the correct response (the fish is always assigned to the left response). Participants were further instructed to use the left and right index fingers for responding and to respond as quickly and accurately as possible.

As target stimuli, we used four colored animal pictures adapted from the Snodgrass and Vanderwarts' pictorial set (Rossion and Pourtois, 2004). Cues were four photographs of human faces (young/old woman, young/old man) from the lifespan database of adult facial stimuli (Minear and Park, 2004) as this task has already been used in a previous aging study (Schmitt et al., 2014a).

In the practice phase, participants performed three blocks of 16 trials each in the following fixed order. The first practice block contained only c-indep trials, followed by a second block of only c-dep trials, which was followed by a third block containing intermixed trials. In the experimental phase, participants performed four intermixed blocks, each containing 41 trials: one start trial, 20 c-indep and 20 c-dep trials. Each block was equal regarding stimulus and response type. Stimulus-response assignments were counterbalanced across participants. The same tasks were used at pre- and post-test, but with different stimulus sets. The testing time was ~15 min.

The trial procedure started with a 250 ms fixation cross, followed by the informative cue, which stayed on the display for 750 ms. After a second 750 ms fixation cross, the target was presented for 3,600 ms and responses had to be executed within the given time window. If participants did not respond within the given time window, the trial was excluded from further analyses. The 500 ms ITI separated two consecutive trials.

#### Training Sessions

The training intervention consisted of eight sessions. Older adults practiced over a time period of 4 weeks with two 45-min sessions per week at Saarland University with the restriction to not train on two consecutive days per week. According to their baseline performance (see above), older participants were assigned to either the single-task training group or the task-switching training group. Both groups received and performed identical tasks (same cues and stimuli) during the training sessions, with the difference that the single-task training group practiced the two tasks (e.g., A and B) in separate blocks, while the task-switching training group only received mixed-task blocks, and by this, practiced the updating of cue information while switching between two tasks.

Each training session consisted of 10 blocks of 41 trials with the block design and trial procedure being identical between the pre- and post-test sessions. In each session, the single-task training group performed five blocks of the one task and five blocks of the other task, whereas the task-switching training group performed 10 mixed-task blocks.

Across the eight training sessions, we used different stimulus material and different tasks given that a previous training study has shown that a variable training lead to greater transfer effects in older adults (Karbach and Kray, 2009). The eight different training tasks were constructed in a way that participants were required to classify pictures according to a semantic or perceptual task (see Table 2). The first four training tasks were adopted from Karbach and Kray (2009) and the other four training tasks were newly created. Stimuli were taken from the databases of Snodgrass and Vanderwart (1980) and Rossion and Pourtois (2004). The order of the eight training tasks was kept constant across the sessions.

**Table 2:** Semantic and perceptual tasks used in the eight training sessions.

**Training session**	**Semantic task (task A)**	**Categories (task A)**	**Perceptual task (task B)**	**Categories (task B)**	**Example stimuli**
Session 1	Transportation	Planes/cars	Number	One/two	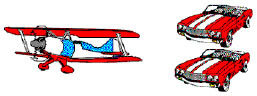
Session 2	Hobby	Music/sports	Color	Blue/orange	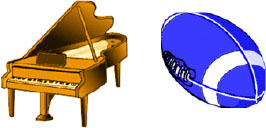
Session 3	Animal	Bird/fish	Direction	Left/right	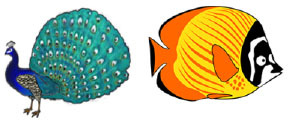
Session 4	Plant	Leaf/flower	Chromaticity	Colored/black- and-white	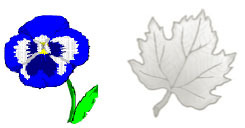
Session 5	Clothing	Hat/shoe	Texture	Dotted/squared	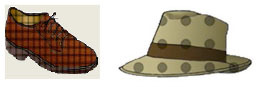
Session 6	Landscape	Building/tree	Orientation	Upright/rotated	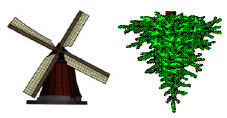
Session 7	Gadget	Toy/tool	Luminance	Bright/dark	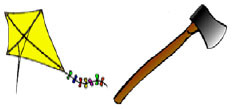
Session 8	Gender	Male/female	Hair-color	Blond/brown	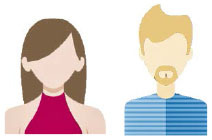

*As in the pretest and posttest sessions, the cues for each the task A and task B referred to the first two letters of the German translation of each task: transformation task (TR = “Transportmittel”; number task (ZA = “Zahl”); hobby task (HO = “Hobby”); color task (FA = “Farbe”); animal task (TI = “Tier”); direction task (RI = “Richtung”); plant task (PF = “Pflanze”); chromaticity task (SÄ = “Sättigung”); clothing task (KL = “Kleidung”); texture task (MU = “Muster”); landscape task (TE = “Terrain”); orientation task (OR = “Orientierung”); gadget task (OB = “Object”); luminance task (LU = “Luminanz”); gender task (GE = “Geschlecht”); hair-color task (HA = “Haarfarbe”)*.

### EEG Recording and Pre-processing

During EEG recording, participants were seated in an electrically shielded and noise-protected room. EEG was recorded using the Brain Vision Recorder Software (Brain Products, Munich, Germany). Fifty-nine Ag/AgCl active Electrodes were attached to elastic caps (Acticap) and arranged in the extended international 10–20 system (Jasper, 1958). Additionally, electro-ocular activity (EOG) was recorded from four electrodes placed at the outer canthi of both eyes and above and below the right eye. The ground electrode was placed at AFz, the online reference at the right mastoid, and impedances were kept below 20 kΩ. EEG data were filtered online with a low-pass filter of 250 Hz and converted analog-to-digital with a sampling rate of 500 Hz. Offline EEG processing was done using EEProbe (ANT). Recordings were band-pass filtered offline from 0.01 to 30 Hz, re-referenced to linked mastoids, and averaged according to the respective experimental conditions. Whenever the standard deviation in a moving 200 ms time interval exceeded 30 μV in ocular electrodes, data were marked as artifacts. These eye movements were corrected by using a linear regression approach (Gratton et al., 1983). All other artifacts in the EEG data were rejected prior to averaging if the standard deviation of the amplitude in a 200 ms interval was above 20 μV in the representative electrode Cz. For the task-switching task, this procedure resulted in the rejection of 43.2 and 46.2% of trials for younger adults, 58.7 and 59.5% for older adults from the single-task training group, and 62.2 and 55.8% for older adults from the task-switching training group, for pre- and post-test, respectively. For the context-updating task, this procedure resulted in the rejection of 33.2 and 31.9% of trials for younger adults, 55.3 and 55.0% for older adults from the single-task training group, and 55.5 and 49.6% for older adults from the task-switching training group, for pre- and post-test, respectively. The rejection rates were similarly distributed for single and mixed trials at pretest and posttest for younger and older adults, and therefore did probably not selectively influence the EEG results. Overall, we made sure that for each condition in each subject, we at least obtained 16 artifact-free trials. Participants that did not fulfill this criterion were excluded from data analyses (see next section).

### Data Analysis

Before analyzing, behavioral performance was screened for extreme values in the baseline and the training data. Mean reaction times (RT) or error rates more than three standard deviations from the corresponding group mean were considered as extreme values which resulted in the exclusion of one older participant from the switching group from all behavioral and ERP analyses. Four participants (two younger adults, one older adult from the single-task training group, and one from the switching group) producing <16 artifact-free trials per task condition were excluded from statistical analyses of the task-switching EEG data. Three participants (two older adults from the single-task training group and one from the switching group) were excluded from the analyses of the AX-CPT EEG data. Data were analyzed with the software package SPSS 24.

For behavioral data analysis of the switching task, the first trial in each block and trials including reaction times (RT) below 100 ms were excluded from the subsequent analyses. Data exclusion was only very minor for the training data (0.16% of the trials in the task-switching group; 0.11% of the trials for the single task group). At pretest, data exclusion was 2.75% for the older group and 0.28% for the younger group, and at posttest, 0.68% for the older group and 0.19% for the younger group). Behavioral results were based on mean RT for correct responses and on error rates.

ERPs were averaged stimulus-locked to the cue and separately for each trial type in time windows from −200 to 1,000 ms (in the switching task) and from −200 to 800 ms (in the AX-CPT) using a 100 ms prestimulus baseline. For both tasks, the cue-locked P3 was analyzed using a mean amplitude measure in a respective time window. The selection of the time windows for statistical analyses was based on the literature and our previous analyses of the cue-locked P3 in these two tasks (e.g., Karayanidis et al., 2003; Kray et al., 2005; Schmitt et al., 2014a), together with the visual inspection of the peaks obtained in the present data. Visual inspection of the cue-locked P3 in the switching task indicated a prolonged P3 component without a clear peak in older adults which is consistent with the literature (for a review, see Gajewski et al., 2018). For this reason, the cue-locked P3 amplitudes were measured in an early 300–500 ms window and a late 500–700 ms window after cue onset and we refrained from analyzing P3 latencies. For the AX-CPT, ERP amplitudes were averaged in different time windows for pretest and posttest due to clear temporal shifts of the peak amplitudes from pretest to posttest in the present data: Cue-locked P3 amplitudes were averaged in a 470–670 ms window at pretest and in a 400–600 ms window at posttest. In line with previous ERP studies, analyses were restricted to the midline electrodes Fz, Cz, and Pz (e.g., Karayanidis et al., 2011). Please note that the trials included in the ERP analyses did not fully correspond to the trials that entered the behavioral analyses.

For all analyses, the alpha level was set to 0.05. Greenhouse–Geisser corrections for non-sphericity (Keselman and Rogan, 1980) were applied when necessary. In this case, epsilon corrected *p*-values are reported together with epsilon values and uncorrected degrees of freedom.

## Results

In the following, we will first report the results of the training data to make sure that participants showed improved task performance during the eight training sessions. Then, we will first analyze age-related differences in the two cognitive control tasks at the behavioral and neuronal level in order to prove whether previous findings on reported age differences can be replicated. Thereafter, we will present the results on the transfer of training to a structural similar switching task at the behavioral and neuronal level. Finally, we will report the results on the transfer to an untrained cognitive control task, also requiring the updating of cue information, as the trained task.

### Training Data

Given that we applied a variable training intervention in which participants performed a new set of tasks in each of the training session, training data will not be analyzed as a function of training session because the eight tasks also differed in task difficulty. Information about training gains in each of the eight sessions is provided in the Supplementary Material. Instead, we determined the training gains within each of the eight training sessions by dividing them into four bins (one bin contains 35 trials) that were then aggregated across the eight sessions (see also Pereg et al., 2013). The corresponding data are shown in Table 3. Mean RTs and error rates were separately analyzed with an ANOVA design, including the between-subjects factor Training Group (task-switching training, single-task training) and the within-subjects factor Bin (1, 2, 3, 4).

**Table 3:** Means (M) and standard deviations (SD) for RTs (ms) and error rates (%) as a function of training group (single task training, task-switching training) and time (bin 1, bin 2, bin 3, bin 4).

	**Time**							
	**Bin 1**		**Bin 2**		**Bin 3**		**Bin 4**	
**Training group**	***M***	***SD***	***M***	***SD***	***M***	***SD***	***M***	***SD***
**RTs (in ms)**								
Single task training	651	78	623	72	611	70	601	69
Task-switching training	735	137	697	134	661	123	647	111
**Error rates (in %)**								
Single task training	2.11	1.04	1.65	1.10	1.60	1.05	1.64	1.07
Task-switching training	6.14	4.10	4.25	2.79	3.18	2.25	2.82	2.24

The results on mean RTs revealed main effects for Bin, *F*_(3,183)_ = 121.82, *p* < 0.001, $\eta_{\text{p}}^{2}$ = 0.67, and Training Group, *F*_(1,61)_ = 6.37, *p* < 0.05, $\eta_{\text{p}}^{2}$ = 0.10, as well as a significant interaction between Bin and Training Group, *F*_(3,183)_ = 11.54, *p* < 0.001, $\eta_{\text{p}}^{2}$ = 0.16. Separate analyses for each training group revealed a linear reduction of mean RTs within training sessions for the task-switching training group, *F*_(1,28)_ = 74.59, *p* < 0.001, $\eta_{\text{p}}^{2}$ = 0.73, as well as for the single-task training group, *F*_(1,33)_ = 78.54, *p* < 0.001, $\eta_{\text{p}}^{2}$ = 0.70. That means, both training groups became continuously faster with increasing practice, and this decrease was larger in the task-switching group than in the single-task training group, while the single-task training group was generally faster in responding (see Table 3).

The results on error rates showed the same pattern. The ANOVA results revealed main effects for Bin, *F*_(3,183)_ = 38.77, *p* < 0.001, $\eta_{\text{p}}^{2}$ = 0.39, and Training Group, *F*_(1,61)_ = 23.27, *p* < 0.05, $\eta_{\text{p}}^{2}$ = 0.28, as well as an interaction between both factors, *F*_(3,183)_ = 21.19, *p* < 0.001 $\eta_{\text{p}}^{2}$ = 0.26. Again, separate analyses for each group indicated a linear reduction of error rates with increasing practice for the task-switching training group, *F*_(1,28)_ = 38.48, *p* < 0.001, $\eta_{\text{p}}^{2}$ = 0.58, that was only marginally significant in the single-task training group, *F*_(1,33)_ = 3.84, *p* = 0.059, $\eta_{\text{p}}^{2}$ = 0.10. In line with the results on mean RTs, both groups became more accurate in responding, while practice effects were larger in the task-switching group than the single-task group, whereas the latter group also produced generally less errors (see Table 3).

### Analysis of Pretest Data

Before analyzing the transfer effects, we looked at the pretest data in order to make sure that we were able to replicate previous results on age differences in the two cognitive control measures, and to examine potential baseline differences between the two training groups. We will report the behavioral and neuronal results first for the switching task, and then for the context-updating task (AX-CPT).

#### Results of the Switching Task at Pretest

To examine age and possible a priori training group differences in task switching, we performed an ANOVA with the between-subjects factor Group (older/single task training, older/task-switching training, younger adults) and the within-subjects factor Trial Type (single, repeat, switch) for the behavioral data that are shown in Table 4. As we were mainly interested in whether younger adults differed from older ones, and whether the older adults training group differed from each other, we conducted an orthogonal group contrasts in the ANOVA design (−2 1 1; 0 −1 1) and focused on interactions with two trial-type contrasts: The first contrast reflects mixing costs (Trial Type Contrast 1: −2 1 1) and the second contrast reflects switching costs (Trial Type Contrast 2: 0 −1 1).

**Table 4:** Means (M) and standard deviations (SD) for RTs (ms) and error rates (%) as a function of group and trial type separately for the pretest and posttest.

	**Younger adults/no training**		**Older adults/single-task training**		**Older adults/task-switching training**	
**Trial type**	***M***	***SD***	***M***	***SD***	***M***	***SD***
**RTs (in ms) at pretest**						
Single	543	90	776	92	743	108
Repeat	611	115	888	132	854	157
Switch	641	122	909	153	882	170
**RTs (in ms) at posttest**						
Single	506	81	693	95	659	94
Repeat	551	117	782	140	714	138
Switch	557	129	817	159	738	166
**Error rates (in %) at pretest**						
Single	2.60	1.61	6.47	4.48	6.96	5.76
Repeat	4.74	3.68	11.38	9.56	10.46	10.78
Switch	4.54	3.51	14.17	7.90	13.51	7.90
**Error rates (in %) at posttest**						
Single	2.92	2.33	4.46	2.57	4.49	2.43
Repeat	6.31	1.78	11.47	6.17	9.33	5.35
Switch	4.04	3.01	10.12	5.75	7.29	4.51

Results for the mean RTs showed reliable mixing costs, *F*_(1,93)_ = 153.76, *p* < 0.001, $\eta_{\text{p}}^{2}$ = 0.63, and switching costs, *F*_(1,93)_ = 27.07, *p* < 0.001, $\eta_{\text{p}}^{2}$ = 0.23. Older adults showed larger mixing costs compared to younger adults, *F*_(1,91)_ = 4.88, *p* < 0.05, $\eta_{\text{p}}^{2}$ = 0.05, but no larger switching costs, *p* = 0.64. Importantly, the two training groups did not significantly differ in the magnitude of mixing and switching costs (*p* = 0.91, *p* = 0.59, respectively).

The analysis of error rates also revealed reliable mixing costs, *F*_(1,93)_ = 48.80, *p* < 0.001, $\eta_{\text{p}}^{2}$ = 0.34, and switching costs, *F*_(1,93)_ = 14.89, *p* < 0.001, $\eta_{\text{p}}^{2}$ = 0.14. For the error rates, older adults showed larger mixing and switching costs than younger adults, *F*_(1,91)_ = 7.47, *p* < 0.01, $\eta_{\text{p}}^{2}$ = 0.08, *F*_(1,91)_ = 9.75, *p* < 0.01, $\eta_{\text{p}}^{2}$ = 0.10, respectively. Again, the two training groups did not differ in mixing and switching costs at pretest (*p* = 0.41, *p* = 0.82, respectively).

**Figure 1:**
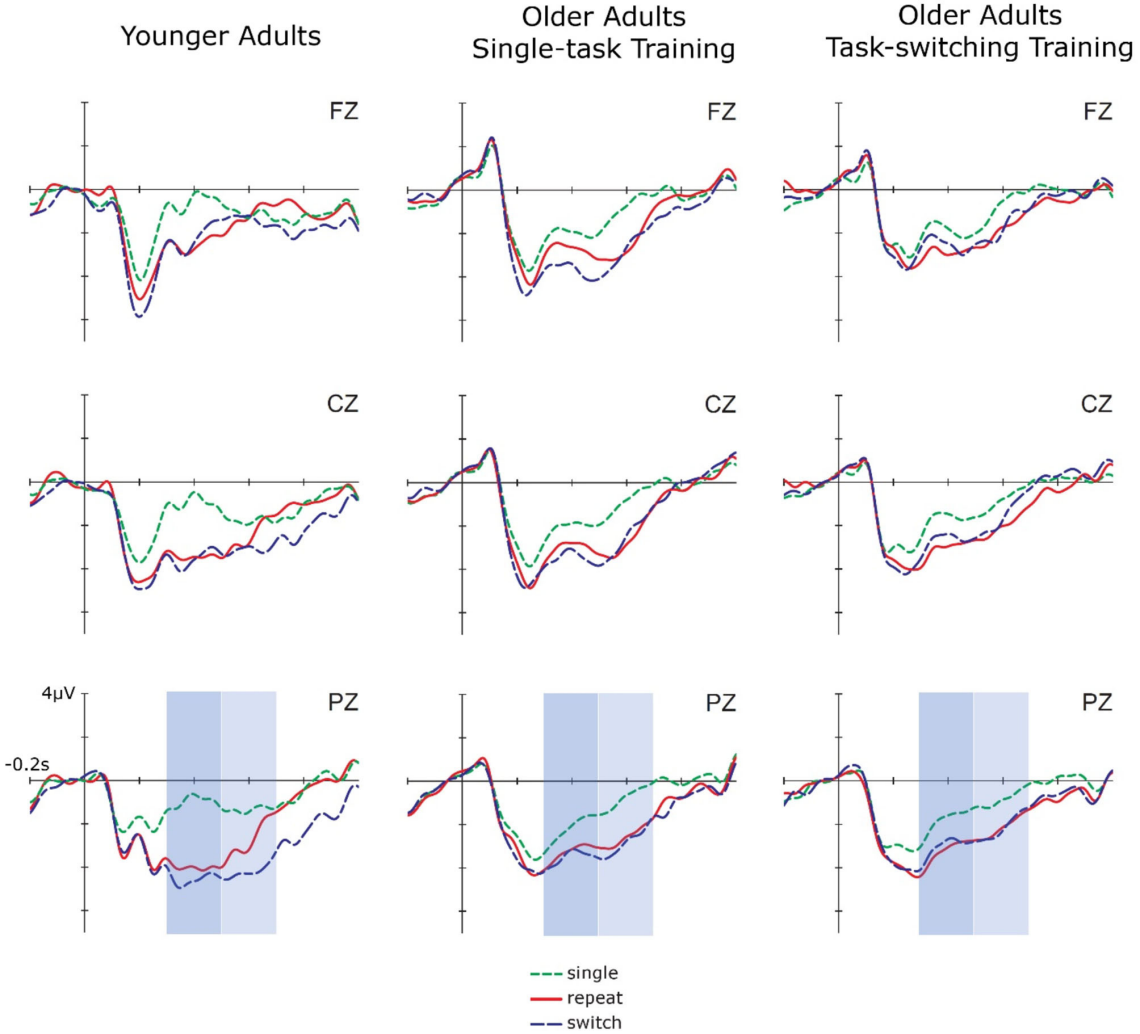
Cue-locked ERPs for single, repeat, and switch trials at pretest for younger adults, older adults in the single-task training, and older adults in the task-switching training group displayed at electrodes Fz, Cz, and Pz.

To examine group differences in early and late cue-locked P3 mean amplitudes in task switching (see Figure 1), we performed ANOVAs with the between-subjects factor Group (older/single, older/switching, younger adults) and the within-subjects factors Trial Type (single, repeat, switch) and Electrode Location (Fz, Cz, Pz), using the same contrasts as before. In addition, to reduce unnecessary comparisons to those of interest, the factor Electrode Location was tested in a repeated contrast (−1 1 0, 0 −1 1).

In early cue-locked P3 amplitudes, this ANOVA yielded a significant Trial Type Contrast 1, *F*_(1,87)_ = 79.65, *p* < 0.01, $\eta_{\text{p}}^{2}$ = 0.48, indicating reliable mixing costs, as well as an interaction between Group and Trial Type Contrast 1, *F*_(2,87)_ = 4.20, *p* < 0.05, $\eta_{\text{p}}^{2}$ = 0.09. *Post-hoc* tests showed that mixing costs were significant in all three groups [younger adults: *F*_(1,28)_ = 60.38, *p* < 0.01, $\eta_{\text{p}}^{2}$ = 0.68; older/single: *F*_(1,32)_ = 10.51, *p* < 0.01, $\eta_{\text{p}}^{2}$ = 0.25; older/switching: *F*_(1,27)_ = 27.41, *p* < 0.01, $\eta_{\text{p}}^{2}$ = 0.50]. However, they were larger for younger adults than for the two older training groups, *mean difference* = 1.19, *SE* = 0.35, *p* < 0.01, while the two older training groups did not differ, *p* = 0.83. Additionally, the three-way interaction between Group, Trial Type Contrast 1, and Electrode Location (Cz vs. Pz) was marginally significant, *F*_(1,87)_ = 2.84, *p* = 0.06, $\eta_{\text{p}}^{2}$ = 0.06. Dissolving this interaction showed that for younger adults, mixing costs in early cue-locked P3 amplitudes differed between electrode locations, *F*_(1,28)_ = 5.38, *p* < 0.05, $\eta_{\text{p}}^{2}$ = 0.16. They were more pronounced at Pz, *F*_(1,28)_ = 95.30, *p* < 0.01, $\eta_{\text{p}}^{2}$ = 0.77, than at Cz, *F*_(1,28)_ = 29.78, *p* < 0.01, $\eta_{\text{p}}^{2}$ = 0.52, which can be inferred from the effect sizes. This interaction was not observed for older adults (all *p* > 0.31).

The same ANOVA was calculated for the late cue-locked P3 amplitudes. This analysis again yielded a significant Trial Type Contrast 1, *F*_(1,87)_ = 38.92, *p* < 0.01, $\eta_{\text{p}}^{2}$ = 0.31, suggesting reliable mixing costs. Mixing costs differed over electrode locations (Cz vs. Pz), *F*_(1,87)_ = 10.46, *p* < 0.01, $\eta_{\text{p}}^{2}$ = 0.27, being larger at parietal, *F*_(1,87)_ = 50.95, *p* < 0.01, $\eta_{\text{p}}^{2}$ = 0.37, than central electrodes, *F*_(1,87)_ = 20.43, *p* < 0.01, $\eta_{\text{p}}^{2}$ = 0.19, which can be inferred from the effect sizes. There was also an interaction between Trial Type Contrast 2 and Electrode Location (Cz vs. Pz), *F*_(1,87)_ = 10.30, *p* < 0.05, $\eta_{\text{p}}^{2}$ = 0.11, which was due to significant switching costs at Pz, *F*_(1,87)_ = 50.95, *p* < 0.01, $\eta_{\text{p}}^{2}$ = 0.37, but not Cz, *p* = 0.92. Although mixing and switching costs did not interact with group, we had hypotheses concerning group differences and thus additionally analyzed each group separately. These analyses demonstrated that for younger adults, the P3 was parietally distributed, *F*_(1,28)_ = 9.83, *p* < 0.05, $\eta_{\text{p}}^{2}$ = 0.26, and mixing, *F*_(1,28)_ = 16.14, *p* < 0.01, $\eta_{\text{p}}^{2}$ = 0.37, as well as switching costs, *F*_(1,28)_ = 5.38, *p* < 0.05, $\eta_{\text{p}}^{2}$ = 0.16, were present at Pz only. In contrast, for older adults mixing costs were still present in this later time interval [older/single: *F*_(1,32)_ = 14.79, *p* < 0.01, $\eta_{\text{p}}^{2}$ = 0.32; older/switching: *F*_(1,27)_ = 33.19, *p* < 0.01, $\eta_{\text{p}}^{2}$ = 0.55] and did not differ between the two groups, *p* = 0.35. Moreover, switching costs were absent (all *p* > 0.49) and there were no effects involving Electrode Location for the older age groups, all *p* > 0.16.

In sum, the behavioral data showed no baseline difference in mixing and switching costs between the two training groups. Age differences were found for mixing costs but not for switching costs at the level of reaction times as well as for mixing and switching costs at the level of errors. P3 amplitudes demonstrated the typical age-related frontal shift in P3 distribution with a clear parietal focus in younger and more evenly distributed P3 in older adults. In the early cue-locked P3, mixing costs were found in all three groups. They were larger for younger adults and the two older groups did not differ from each other. In the later cue-locked interval, switching costs emerged for younger adults, while for older adults mixing costs extended into this interval and switching costs were not present.

#### Results of the Context-Updating Task at Pretest

To examine age and group differences in context updating, we performed an ANOVA with the between-subjects factor Group (older/single task training, older/task-switching training, younger adults) and the within-subjects factor Trial Type (c-dep, c-indep), using the same group contrasts as before. The behavioral data are shown in Table 5.

**Table 5:** Means (M) and standard deviations (SD) for RTs (ms) and error rates (%) as a function of group and trial type separately for the pretest and posttest in the AX-CPT.

	**Younger adults/no training**		**Older adults/single task training**		**Older adults/task-switching training**	
**Trial type**	***M***	***SD***	***M***	***SD***	***M***	***SD***
**RTs (in ms) at pretest**						
c-indep	477	66	717	173	655	125
c-dep	572	124	952	159	911	222
**RTs (in ms) at posttest**						
c-indep	429	66	614	118	555	106
c-dep	498	144	801	229	702	188
**Error rates (in %) at pretest**						
c-indep	1.23	1.50	4.92	11.74	1.33	2.25
c-dep	3.77	3.00	15.06	12.36	11.73	12.40
**Error rates (in %) at posttest**						
c-indep	0.76	1.23	2.72	5.98	0.84	1.75
c-dep	3.64	2.25	11.77	14.08	6.36	7.09

Results indicated a reliable context effect for reaction times, *F*_(1,93)_ = 129.94, *p* < 0.001, $\eta_{\text{p}}^{2}$ = 0.58, and for error rates, *F*_(1,93)_ = 45.73, *p* < 0.001, $\eta_{\text{p}}^{2}$ = 0.33, that is, better performance in c-indep than c-dep trials. For both measures, older adults showed a greater context effect compared to younger adults, *F*_(1,91)_ = 20.85, *p* < 0.001, $\eta_{\text{p}}^{2}$ = 0.19 and *F*_(1,91)_ = 11.13, *p* = 0.001, $\eta_{\text{p}}^{2}$ = 0.11, respectively, but again the two training groups did not significantly differ from each other (*p* = 0.60, *p* = 0.92, respectively).

**Figure 2:**
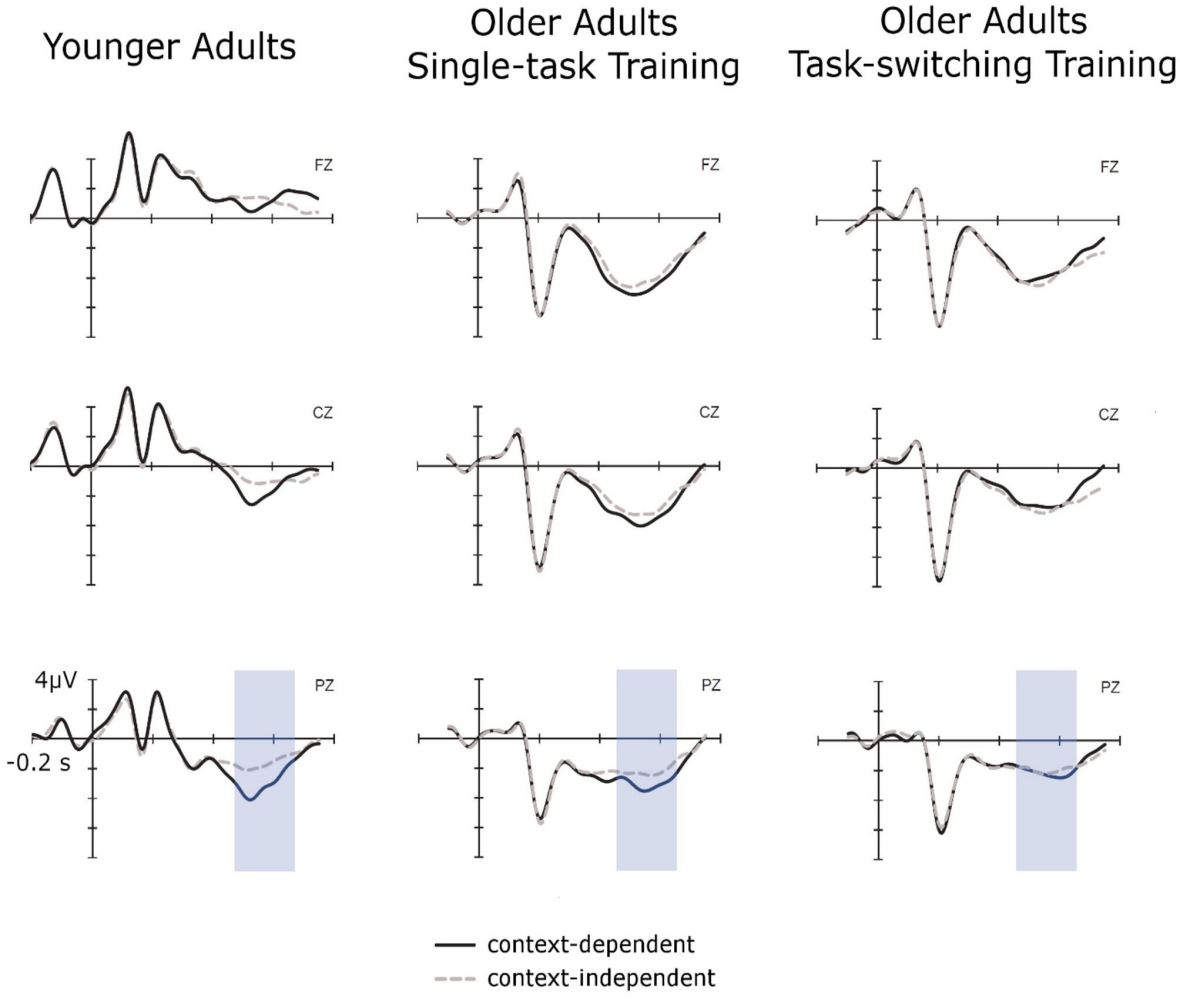
Cue-locked ERPs for context-independent and context-dependent trials at pretest for younger adults, older adults in the single-task training group, and older adults in the task-switching training group displayed at electrodes Fz, Cz, and Pz.

For the analysis of the cue-locked P3 amplitudes (see Figure 2) in the context updating task, we applied the same ANOVA design as for the behavioral data with the additional factor Electrode Location (Fz, Cz, Pz). Again, to reduce unnecessary comparisons to those of interest, the factor Electrode Location was tested in a repeated contrast (−1 1 0, 0 −1 1).

Cue-locked P3 amplitudes displayed an interaction between Group and Electrode [Fz/Cz: *F*_(2,88)_ = 54.70, *p* < 0.01, $\eta_{\text{p}}^{2}$ = 0.55; Cz/Pz: *F*_(2,88)_ = 5.73, *p* < 0.01, $\eta_{\text{p}}^{2}$ = 0.12], which was due to a clear parietal topography of the P3 for younger adults [Fz < Cz: *F*_(1,30)_ = 84.95, *p* < 0.01, $\eta_{\text{p}}^{2}$ = 0.74; Cz < Pz: *F*_(1,30)_ = 17.84, *p* < 0.01, $\eta_{\text{p}}^{2}$ = 0.37], but a frontal topography for both groups of older adults [Fz > Cz for older/single, *F*_(1,31)_ = 11.59, *p* < 0.01, $\eta_{\text{p}}^{2}$ = 0.27, and older/switching, *F*_(1,27)_ = 41.12, *p* < 0.01, $\eta_{\text{p}}^{2}$ = 0.60, respectively]. Additionally, the factor Trial Type showed interactions with Group, *F*_(2,88)_ = 3.22, *p* < 0.05, $\eta_{\text{p}}^{2}$ = 0.07, and with Electrode (Cz/Pz), *F*_(1,88)_ = 5.23, *p* < 0.05, $\eta_{\text{p}}^{2}$ = 0.06. In younger adults, a clear context effect emerged with P3 amplitudes for c-indep trials being smaller than those for dependent trials, *F*_(1,30)_ = 8.80, *p* < 0.01, $\eta_{\text{p}}^{2}$ = 0.23. Context effects were not present for older adults from the single-task group, *p* = 0.10. Older adults from the switching group displayed a marginally significant interaction between Trial Type and Electrode Location (Cz/Pz), *F*_(1,27)_ = 3.34, *p* = 0.09, $\eta_{\text{p}}^{2}$ = 0.10, but follow-up test at Cz and Pz did not result in any significant context effects, all *p* > 0.37.

In sum, we found a larger context effect in older than in younger adults for reaction times and error rates while both training groups did not differ in the magnitude of the context effect at pretest. The cue-locked P3 displayed a clear parietal topography in younger adults and was shifted toward frontal electrodes in both groups of older adults. Moreover, there was a P3 context effect present in younger adults, but absent for both groups of older adults.

### Transfer of Training to an Untrained Switching Task

#### Transfer at the Behavioral Level

To examine the transfer of training in task switching to a similar switching task, we focused the analysis on reaction times, as previous studies usually did not find transfer at the level of error rates, mostly because error rates are relatively small and there is less room for improvement. To determine relative improvements from pretest to posttest (taken into account age-group differences at pretest), we also analyzed log-transformed RTs. Note that the difference between log-transformed variables corresponds to a proportional score.

**Figure 3:**
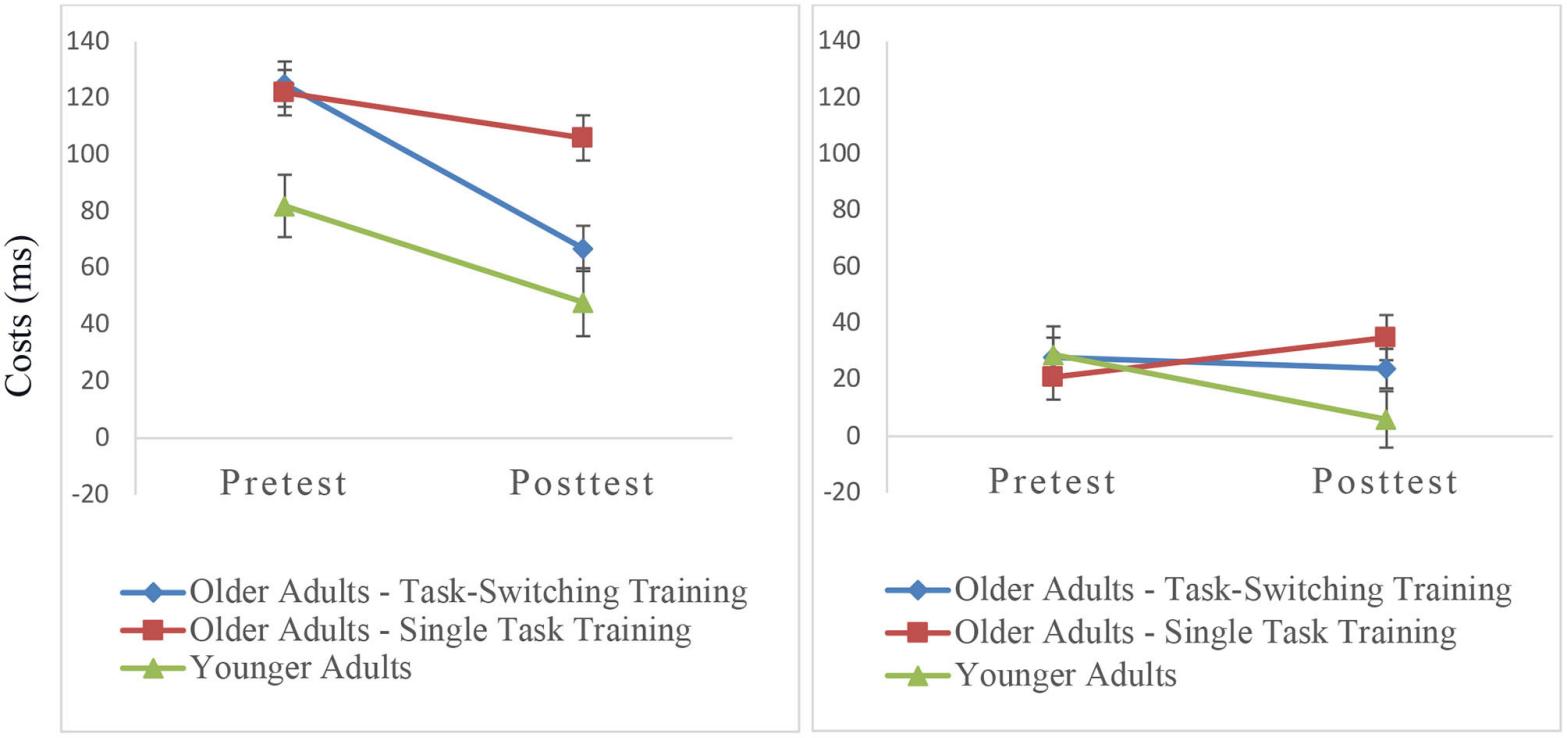
Mixing costs **(left)** and switching costs **(right)** on the level of mean RT as a function of group and session. Error bars refer to standard errors of the mean.

We calculated an ANOVA with the within-subjects factors Session (pretest, posttest) and Trial Type (single, repeat, switch) and the between-subjects factor Group (older/single task training, older/task-switching training, younger adults). As can be seen in Figure 3, there was a relative reduction in mixing costs from pretest to posttest, *F*_(1,93)_ = 11.34, *p* < 0.001, $\eta_{\text{p}}^{2}$ = 0.11, with the task-switching group showing a larger improvement than the single task training group from pretest to posttest, *F*_(1,91)_ = 4.04, *p* < 0.05, $\eta_{\text{p}}^{2}$ = 0.04. Moreover, the difference in mixing costs between both groups was significant at posttest, *F*_(1,91)_ = 4.51, *p* < 0.05, $\eta_{\text{p}}^{2}$ = 0.05, while the magnitude of mixing costs between the older task-switching training group and the younger control group was not significant (*p* = 0.39). We also obtained group differences in relative improvements of switching costs namely a larger reduction of switching costs in the younger control group than in the two training groups, *F*_(1,91)_ = 9.30, *p* < 0.01, $\eta_{\text{p}}^{2}$ = 0.09.

#### Transfer at the Neuronal Level

**Figure 4:**
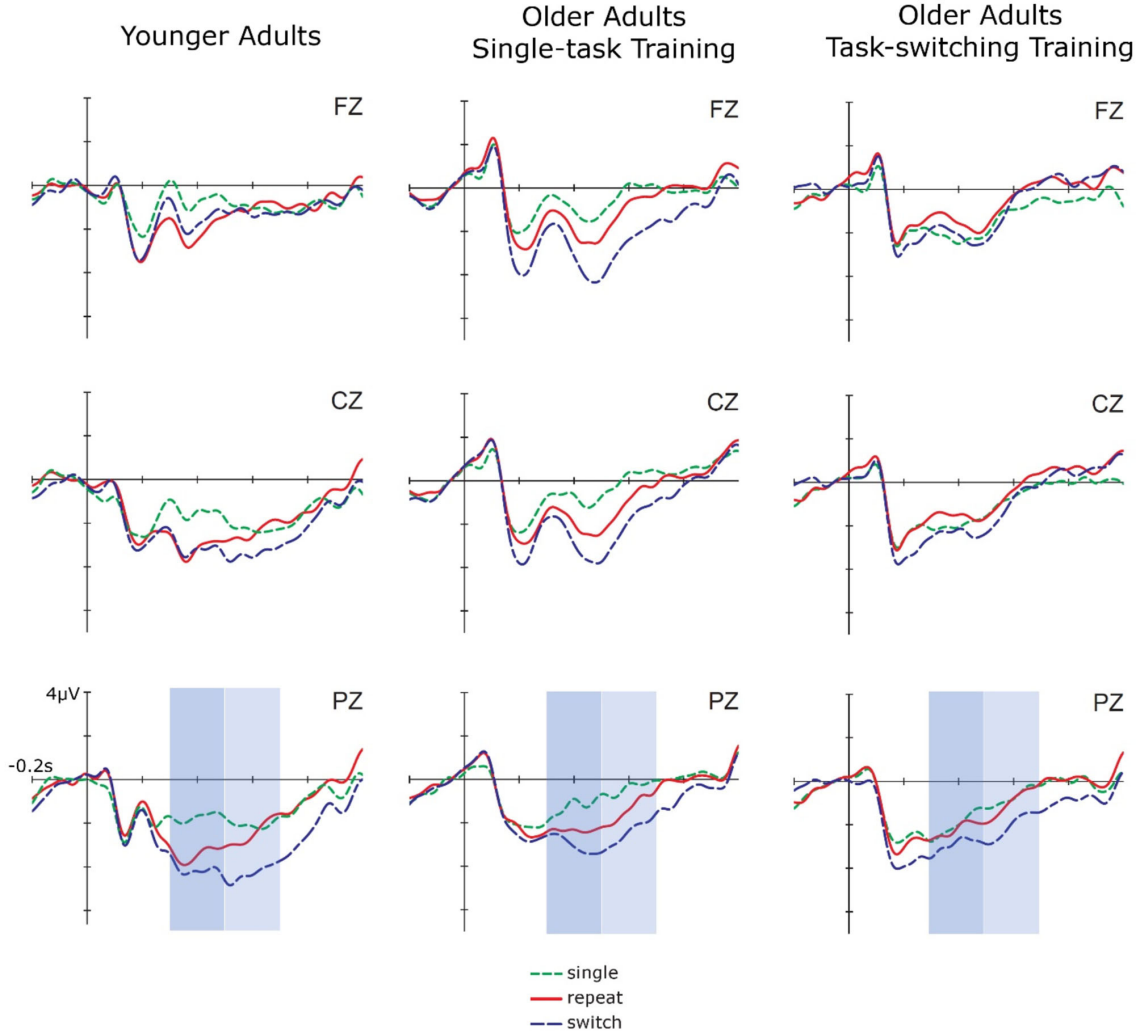
Cue-locked ERPs in single, repeat, and switch trials at posttest for younger adults, older adults in the single task training group, and older adults in the task-switching training group displayed at electrodes Fz, Cz, and Pz.

**Figure 5:**
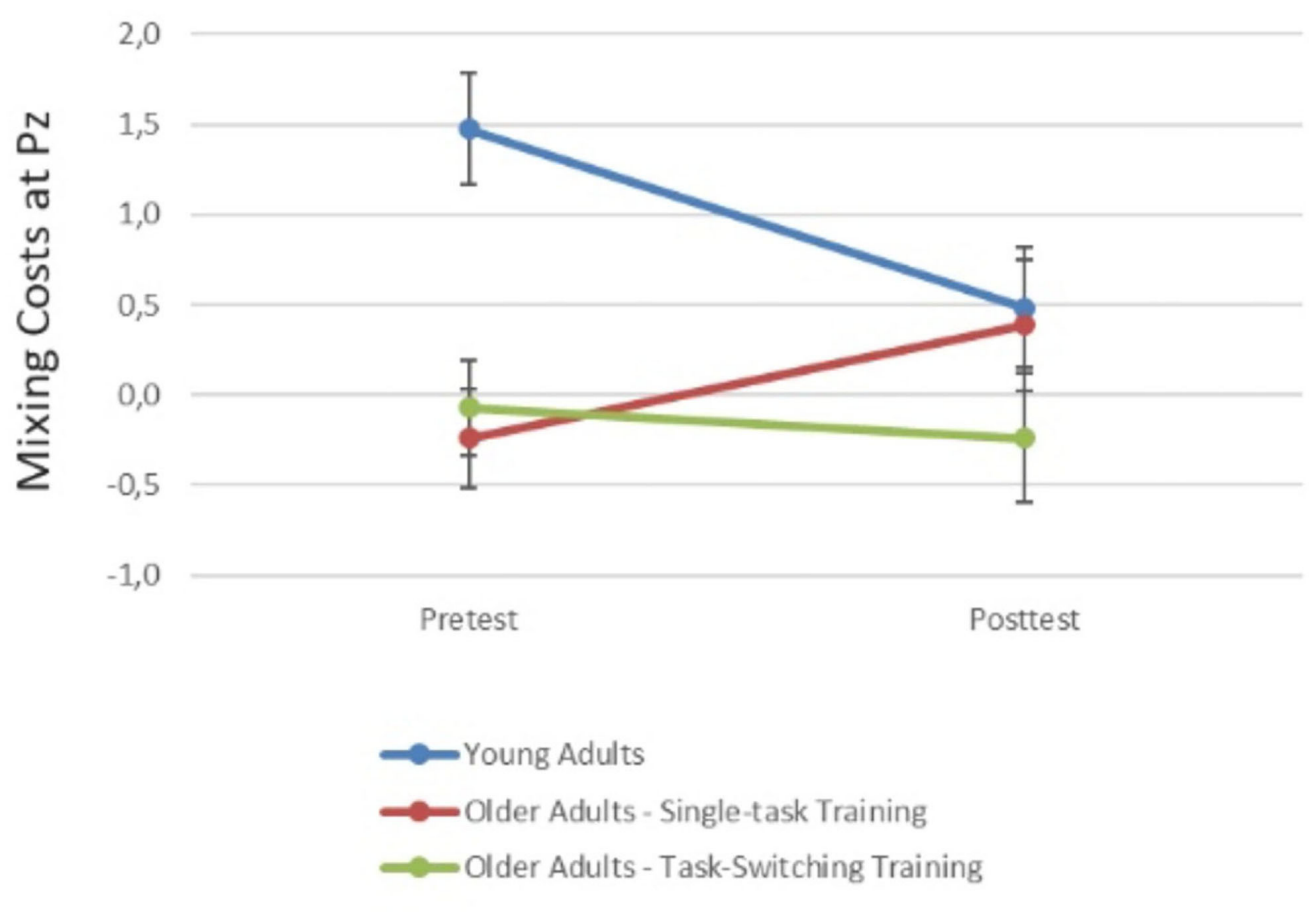
Mixing costs (calculated as the difference between single trials and the mean of repeat and switch trials) in early cue-locked P3 amplitudes at electrode Pz for pre- and post-test. Smaller values reflect smaller mixing costs. Error bars refer to standard errors of the mean. Mixing costs seem to decrease from pre- to post-test for younger adults and older adults from the task-switching training group, while they show a tendency to increase for older adults from the single-task training group.

To examine near transfer to an untrained task-switching task in early and late cue-locked P3 mean amplitudes (see Figure 4), we performed ANOVAs with the between-subjects factor Group (older/single task training, older/task-switching training, younger adults) and the within-subjects factors Session (pretest, posttest), Trial Type (single, repeat, switch), and Electrode Location (Fz, Cz, Pz), using the same contrasts as before (see Results of the Switching Task at Pretest) and focusing on effects including the factor Session. For the early cue-locked P3 amplitude, this ANOVA yielded a significant four-way interaction between Group, Session, Trial Type Contrast 1, and Electrode Location (Cz/Pz), *F*_(1,87)_ = 3.99, *p* < 0.05, $\eta_{\text{p}}^{2}$ = 0.08. For younger adults, there was a tendency for mixing costs to change over sessions, *F*_(1,28)_ = 3.86, *p* = 0.06, $\eta_{\text{p}}^{2}$ = 0.12, with mixing costs being larger at pretest, *F*_(1,28)_ = 60.38, *p* < 0.01, $\eta_{\text{p}}^{2}$ = 0.68, than at posttest, *F*_(1,28)_ = 16.21, *p* < 0.01, $\eta_{\text{p}}^{2}$ = 0.37, as can be inferred from the effect sizes. Moreover, for younger adults the topography (Fz/Cz) of the P3 changed over sessions, *F*_(1,28)_ = 7.76, *p* < 0.01, $\eta_{\text{p}}^{2}$ = 0.22. While there was no difference between frontal and central electrodes at pretest, *p* = 0.21, there was a clear difference at posttest, *F*_(1,28)_ = 12.49, *p* < 0.01, $\eta_{\text{p}}^{2}$ = 0.31, indicating less frontal involvement. For older adults from the single-task group, there was also a tendency for mixing costs to change from pretest to posttest, *F*_(1,38)_ = 3.07, *p* = 0.09, $\eta_{\text{p}}^{2}$ = 0.09, but with mixing costs being smaller at pretest, *F*_(1,32)_ = 10.51, *p* < 0.01, $\eta_{\text{p}}^{2}$ = 0.25, than at posttest, *F*_(1,32)_ = 23.27, *p* < 0.01, $\eta_{\text{p}}^{2}$ = 0.42 (see effect sizes). For older adults from the switching group, mixing costs marginally changed with Session and Electrode (Cz/Pz), *F*_(1,27)_ = 3.78, *p* = 0.06, $\eta_{\text{p}}^{2}$ = 0.12. While mixing costs were present at Cz, *F*_(1,27)_ = 20.61, *p* < 0.01, $\eta_{\text{p}}^{2}$ = 0.43, and Pz, *F*_(1,27)_ = 23.25, *p* < 0.01, $\eta_{\text{p}}^{2}$ = 0.46, at pretest, they were only present at Pz at posttest, *F*_(1,27)_ = 9.79, *p* < 0.01, $\eta_{\text{p}}^{2}$ = 0.27(Cz: *p* = 0.66). Moreover, from the effect sizes it can be inferred that mixing costs were smaller at posttest as compared to pretest (see Figure 5).

For the late cue-locked P3 amplitude, this ANOVA resulted in a marginally significant four-way interaction between Group, Session, Trial Type Contrast 1, and Electrode Location (Cz/Pz), *F*_(2,87)_ = 3.01, *p* = 0.05, $\eta_{\text{p}}^{2}$ = 0.07, and a significant interaction between Session and Trial Type Contrast 2, *F*_(1,87)_ = 4.32, *p* < 0.05, $\eta_{\text{p}}^{2}$ = 0.05. For younger adults and for older adults from the single-task group, there was no significant interaction including Session and Trial Type Contrast 1, all *p* > 0.23. In contrast, for older adults from the switching group, there was an interaction between Session and Trial Type Contrast 1, *F*_(1,27)_ = 5.15, *p* < 0.05, $\eta_{\text{p}}^{2}$ = 0.16, and a marginally significant interaction between Session, Trial Type Contrast 1, and Electrode (Cz/Pz), *F*_(1,27)_ = 3.16, *p* = 0.09, $\eta_{\text{p}}^{2}$ = 0.11. In this group, mixing costs were present at Cz, *F*_(1,27)_ = 21.47, *p* < 0.01, $\eta_{\text{p}}^{2}$ = 0.44, and Pz, *F*_(1,27)_ = 17.59, *p* < 0.01, $\eta_{\text{p}}^{2}$ = 0.39, at pretest, but only at Pz, *F*_(1,27)_ = 7.65, *p* < 0.05, $\eta_{\text{p}}^{2}$ = 0.22, at posttest (Cz: *p* = 0.89). Again, as for the early cue-locked P3, effect sizes indicate that mixing costs were smaller at posttest as compared to pretest. As for switching costs, they were not significant at pretest (*p* = 0.55), but reliable at posttest, *F*_(1,87)_ = 14.90, *p* < 0.01, $\eta_{\text{p}}^{2}$ = 0.15.

In sum, older adults from the switching group showed a reduction in behavioral mixing costs to the level of (untrained) younger adults. Improved behavioral switching costs were also found for younger adults at posttest. In cue-locked P3 amplitudes, a reduction in mixing costs was found for younger adults and for older adults from the switching group. Additionally, the switching group also showed a parietally focused P3 topography after training, i.e., it became similar to the younger adults' P3. For all groups, switching costs emerged post-training.

### Transfer of Training to an Untrained Context Updating Task (AX-CPT)

#### Transfer at the Behavioral Level

To examine the transfer of training in task switching to the untrained AX-CPT, we also focused the analysis on reaction times and we controlled for relative improvements from pretest to posttest by also analyzing log-transformed RTs.

**Figure 6:**
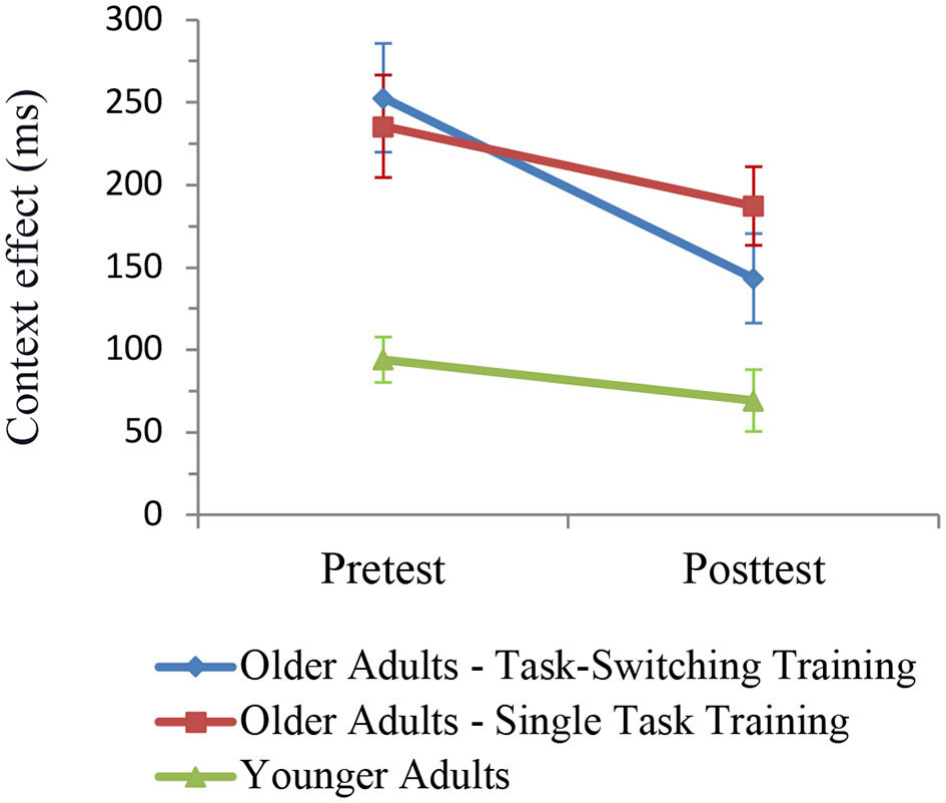
Context effects (c-dep–c-indep) on the level of mean RT as a function of group and session. Error bars refer to standard errors of the mean.

The ANOVA included the within-subjects factors Session (pretest, posttest) and Trial Type (c-indep, c-dep) and the between-subjects factor Group (older/single task training, older/task-switching training, younger adults). The results are shown in Figure 6. All groups showed a relative reduction in the context effect from pretest to posttest, *F*_(1,93)_ = 10.35, *p* < 0.01, $\eta_{\text{p}}^{2}$ = 0.10. There was only a tendency that the task-switching group showed a larger improvement than the single-task training group, that is, a greater reduction in the context effect, *F*_(1,91)_ = 2.94, *p* = 0.09, $\eta_{\text{p}}^{2}$ = 0.03. However, the difference in the context effect between the task-training group and the single-task training group was not significant at posttest, *p* < 0.21, $\eta_{\text{p}}^{2}$ = 0.05.

#### Transfer at the Neuronal Level

**Figure 7:**
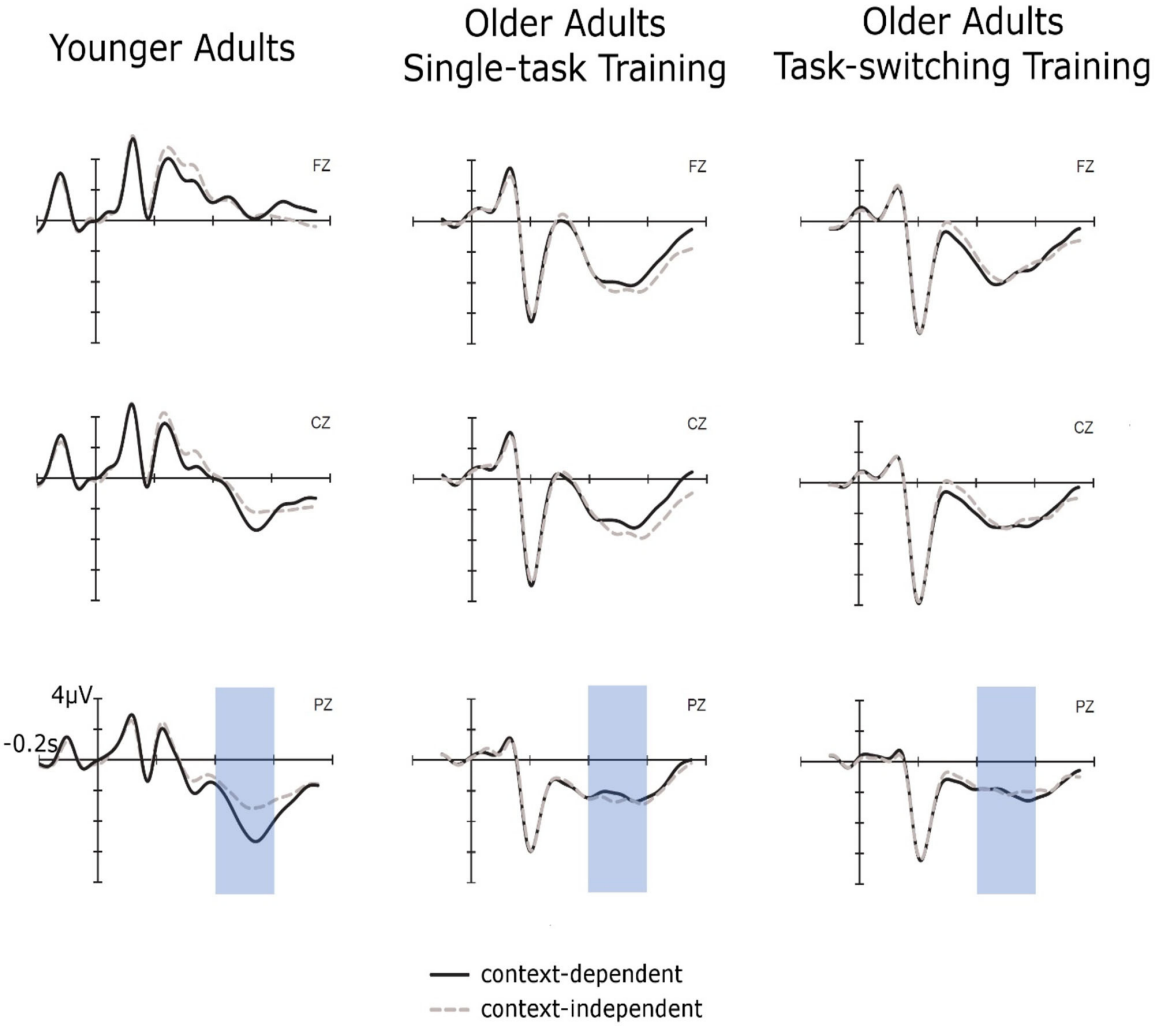
Cue-locked ERPs for context-dependent and context-independent trials at posttest for younger adults, older adults in the single-task training group, and older adults in the task-switching training group displayed at electrodes Fz, Cz, and Pz.

To examine near transfer to an untrained context-updating task in cue-locked P3 mean amplitudes (see Figure 7), we performed an ANOVA with the between-subjects factor Group (older/single task training, older/task-switching training, younger adults) and the within-subjects factors Session (pretest, posttest), Trial Type (c-dep, c-indep), and Electrode Location (Fz, Cz, Pz), using the same contrasts as before (see Results of the Context-Updating Task at Pretest) and focusing on effects including the factor Session. This ANOVA did not yield any effect including the factor Session (all *p* > 0.23), indicating no training-induced changes and no changes in age-related differences.

In sum, we obtained no training-specific changes in the context effect or the cue-P3. Thus, neither the behavioral data nor the neuronal data provided convincing evidence for transfer of training in updating in a switching task to updating of cue information in the modified AX-CPT.

## Discussion

The primary aim of this study was to examine whether an intensive task-switching training would improve older adults' early task-preparatory processes, such as updating higher-order task rules in advance in an untraining switching task, and whether it would transfer to another control task also requiring the updating of cue and rule information. To this end, we applied a cued-based switching task and a context updating task at pretest and posttest and measured changes in task performance and neuronal correlates of cue updating, the cue-P3, in older as well as in younger adults. Only older adults received an intensive variable training in which new tasks and stimuli were presented in each of the eight training sessions. Training-specific effects were determined by comparing a task-switching training group and a single-task training group. The task-switching training group worked through a task-switching training with two tasks changing at random in mixed blocks, thus supposedly training cue updating. The single-task training group also performed both tasks but in separate blocks, so that the task cue was redundant and updating unnecessary.

First, the results of our study revealed that both groups of older adults showed improving task performance over the eight training sessions and that these improvements were larger for the task-switching than for the single-task training group. At pretest, we also found age differences in cognitive control measures that were generally in line with those reported in the literature, namely larger mixing costs (task switching task) and context effects (context updating task) in older than in younger adults on the behavioral level (in reaction times and error rates; Kray and Ferdinand, 2014; Schmitt et al., 2014a) as well as on the neuronal level (in cue-P3; Eppinger et al., 2007; Friedman et al., 2008; Whitson et al., 2014). In addition, we found the commonly reported age-related frontal shift in cue-P3 amplitudes in the task-switching (Kray et al., 2005; Eppinger et al., 2007; Karayanidis et al., 2011; Gajewski et al., 2018) and the context updating task (Schmitt et al., 2014a,b), which is often interpreted in terms of compensation: Older adults need to recruit frontal brain areas to a larger extent in order to keep their performance up (Friedman, 2008; Reuter-Lorenz and Cappell, 2008). In the behavioral data of the task-switching task, we additionally obtained switching costs which were larger for younger than for older adults. Switching costs also emerged in the late cue-P3, but only for younger adults (Eppinger et al., 2007; Friedman et al., 2008; Karayanidis et al., 2011; Whitson et al., 2014). Importantly, both training groups of older adults did not differ in performance nor in the amplitude or topography of the cue-P3 at pretest, thus there were no baseline differences between the two older training groups.

Second, we examined potential transfer of training in task switching to an untrained switching task. On the behavioral level, we found that the task-switching group showed a larger reduction in mixing costs compared to the single task group. Moreover, at posttest, the magnitude of mixing costs did not differ between the task-switching group and the young control group but was smaller in comparison to the single-task group. Thus, only the intensive task-switching training of the older task-switching group resulted in the disappearance of age effects in behavioral mixing costs. In line with the behavioral results, in the cue-P3, younger adults showed a tendency for decreased mixing costs (cf. Küper et al., 2017) and younger as well as older adults from the switching group showed less involvement of frontal brain areas after the training. This means that a) the training resulted in more focused P3 topographies for younger and older adults, and b) that after the task-switching training, older adults' cue-P3 topography became more similar to younger adults' P3 topography, i.e., the usually found frontal P3 shift which probably indicates compensation mechanisms in older adults was diminished. In contrast, for the older adults from the single-task training group, mixing costs were even larger at posttest. By this, our results suggest that older adults can be trained in applying proactive control (here updating of task rules after cue presentation).

Together, these findings support the notion of a training-specific transfer to a similar but untrained switching task. According to the classic view of the P3b (Donchin, 1981; Donchin and Coles, 1988; Polich, 2007), one could argue that the process reflecting updating of the upcoming task rules has become more efficient after training in older adults because improvements in task switching seem to be due to updating being applied in those trials where it is actually needed. This idea also matches the result of emerging switching costs in posttest (although this effect was not specific to the switching group). These findings may also explain why a training that is less specific as for the processes trained (e.g., the cognitive training used by Küper et al., 2017) does only result in a general and unspecific improvement in updating processes, but not in more efficient updating in the sense of being able to apply it specifically in those situations where it is needed. Additionally, our results demonstrate that training in an easier task (the single-task training) that does not support the necessary cognitive processes (here, the preparatory updating processes that are trained in mixed blocks) may also hinder performance in a more difficult (switching) task.

Third, there was no transfer to a different cognitive control task, that is, we found no training-specific improvements in updating cue-relevant information in the AX-CPT, neither at the behavioral nor at the neuronal level. Although there was a tendency that the behavioral context effect was more strongly reduced in the task-switching group than in the single-task group, the magnitude of the context effect at posttest did not differ across groups. Moreover, the P3 amplitudes of context-dependent and context-independent trials of the older groups were nearly fully overlapping (in contrast to younger adults) with no changes from pretest to posttest. The lack of transfer in the efficiency of updating task-relevant information is surprising, given the similarity across both tasks and the assumed underlying processes involved. A possible explanation, however, could be the differences in task cues between the two tasks. While symbolic cues, namely the first two letters of the semantic category of a task rule, were used in the switching task, the cues in the AX-CPT were more arbitrary, namely pictures of young and old men and women that cued several very specific if-then rules. Thus, the symbolic cues are most likely much easier translated into the actual task goal as compared to the pictorial cues. This also means that the training of cue updating in the switching task was rather specific (letters) and did not generalize to other kinds of cues (pictures) indicating the need for updating. Another difference across the two tasks is that in the switching task cues (letters) and targets (pictures) are clearly separable which is not the case in the AX-CPT in which cues (pictures of persons) and targets (pictures of animals) are more similar. Furthermore, the response conflict was higher in the AX-CPT as four different types of S-R rules had to be performed repeatedly and in context-dependent trials the S-R rule was reversed. In contrast, in the switching task, participants performed two task sets, meaning that a class of stimuli was linked to a response depending on goals and not a specific kind of stimulus. Hence, although at first glance both tasks and paradigms have some aspects in common, such as requiring updating and switching between rules, they also differ in crucial aspects, such as cue translation, type of stimuli, and the amount of response conflict, which might all hamper transfer. The present findings also correspond to a previous finding from one of our training studies in which older adults were trained in using a verbal self-instruction, that is, they were told to name aloud the next task during the preparation interval (while no task cues were present). Interestingly, such verbal self-instructions are very useful in improving switching performance (i.e., reducing mixing costs as well as age differences therein; Kray et al., 2008). However, they were not easily transferred to a new switching situation requiring updating of new task goals and by this new verbal self-instruction rules (Karbach et al., 2010). Hence, transfer of training in updating processes seems rather narrow and limited to a specific type of switching situation.

Interestingly, there is also evidence suggesting that the updating process used to prepare for the upcoming task is not a unitary process (for a review, see Karayanidis and Jamadar, 2014), but varies with different task characteristics. Important for the present study is a finding by Nicholson et al. (2006), who found distinct early and late aspects of the cue-P3 that were related to specific aspects of switch preparation in a task-switching paradigm. Particularly, they found that cues indicating a switch to a new task rule (called switch-to cues) elicited an early and a late cue-P3, while cues signaling switch without informing about what task participants will have to conduct (termed switch-away cues) did only elicit the early aspect of the cue-P3. Nicholson et al. (2006) therefore argued that the early aspect of the cue-P3 relates to disengagement from the irrelevant task or an activation of the intention to shift, while the later cue-P3 indexes the actual reloading of the relevant task. This distinction might be of great importance for the present study because they might contribute to explain the lack of transfer from our task-switching task to the context updating task. In the task-switching task used here, a cue always signaled the need to switch as well as the task rule participants would have to switch to. This was not so easy to differentiate in the context updating task where a cue could be non-informative (context-independent trials) or inform about possible tasks to execute after target presentation. Thus, the findings of Nicholson et al. (2006) further corroborate our *post-hoc* speculation that the updating processes that were trained in the task-switching training are very specific to the task at hand and not necessarily exactly the same updating processes that are needed in the context updating task.

A first limitation of the present study is the selectivity of the older sample. In general, the older participants who took part in this study were very healthy and motivated (they were able to come to the university for 10 sessions, mostly with their own car, and also took part in the fMRI part of the study). This means, that their performance probably was in the upper range of their age group and they had less room for training-related improvement. However, this was true for both groups of older adults, but still both groups showed very different patterns in the near transfer to the untrained switching task. Therefore, it is very unlikely that it is the reason for the lack of transfer to the context-updating task. A second limitation is that the older single-task training group also had at least some experience in switching, because both types of tasks had to be performed in one training session. Nevertheless, it is unlikely that this influenced the present pattern of results, as the single-task group showed clear differences from the switching group and even a slightly negative transfer to the untrained switching task. A third limitation is that the study also included an additional fMRI session at pretest for a smaller subsample of older adults (*n* = 25 for the task-switching group and *n* = 25 for the single-task group) that might already have induced fast learning effects in performing the switching tasks. Indeed, the behavioral mixing costs were already quite low at the fMRI pretest session (71 ms for the single task group and 61 ms for the single task group) which could either be due to the smaller and selective subsample or due to differences in the trial procedure of the switching task that needed to be adapted for the event-related fMRI design. Although task-cue and target presentation times were identical, the time intervals after responding were partly longer in the fMRI design (for details, see Dörrenbächer et al., 2020). Nevertheless, although improvements during training under variable training conditions are sometimes absent, transfer effects can occur and can be larger compared to identical training conditions (practice the same switching task across sessions) as older adults are trained in adapting to new updating situations (see Karbach and Kray, 2009). This also means that the amount or the presence of training improvements is not always a precondition for the occurrence of transfer effects as it depends on the type and complexity of the training situations. More important probably is whether the training situation is demanding, and by this induces a mismatch between an individual's actual performance and the demands of the training task. Here the mismatch is induced by a variable set of tasks participants had to perform in each of the training session but can be also induced by adaptive training procedures (see Lövdén et al., 2010), which needs to be considered in the planning of training studies. Finally, although behavioral task-specific transfer effects were small in the fMRI study, we found evidence for neuronal transfer (Dörrenbächer et al., 2020). In this study, we applied a hybrid fMRI design that allowed to examine training-related changes in spatial-temporal brain activation changes. In line with the reduction of mixing costs in the cue-P3 in the present study, we found training-specific changes in brain activations for the cue-related time interval, namely a selective reduction in brain activation in the bilateral mid ventro-lateral prefrontal cortex and the left inferior frontal junction (IFJ) that are known to be involved in maintaining and top-down biasing of task-set representations, and by this support proactive task preparation. Future studies need to clarify whether the observed training-related changes in the cue-P3 in the present study are related to these brain activation changes.

To conclude, our results revealed that older adults who were trained in cue updating show training-specific improvements in preparatory processes during task switching. These improvements were mainly visible in a reduction of behavioral mixing costs and a reduction of mixing costs in the cue-related P3, indicating an improvement specifically in preparatory updating processes. Additionally, the topography of the cue-P3 changed with training from a very broad to a parietally focused scalp distribution closely resembling those in younger adults. However, transfer of the training to context-updating processes in the untrained AX-CPT were not obtained, neither at the behavioral nor at the neuronal level. These results demonstrate that transfer of training updating processes is rather narrow and limited to a specific type of switching situation.

## Data Availability Statement

The raw data supporting the conclusions of this article will be made available by the authors, without undue reservation.

## Ethics Statement

The studies involving human participants were reviewed and approved by Ethikkommission der Fakultät HW, Universität des Saarlandes, Campus A1.3, 66123 Saarbrücken. The patients/participants provided their written informed consent to participate in this study.

## Author Contributions

KS did the pre-processing of the behavioral and EEG data, run the analyses and wrote the first draft of the methods and the results part. JK re-run the behavioral analyses. NF re-run the statistical analyses of the ERP data and wrote the corresponding parts in the introduction, methods, results, and discussion. All authors contributed to the article and approved the submitted version.

## Conflict of Interest

The authors declare that the research was conducted in the absence of any commercial or financial relationships that could be construed as a potential conflict of interest.
