# Supplementary material for: Training and Transfer of Cue Updating in Older Adults Is Limited: Evidence From Behavioral and Neuronal Data
Source: Front Hum Neurosci. 2020 Dec 4;14:565927. doi: 10.3389/fnhum.2020.565927 (PMC7746801; doi:10.3389/fnhum.2020.565927)
Supplement: Supplementary file 1 [file Table_1.pdf]

Supplementary material

Training gains (as difference in performance of mean RTs between Bin 4 and Bin 1) in each of the eight training sessions for the two training groups are shown in Figure 1. Due to differences in task difficulty, training gains varied across sessions. In particular, in the task-switching training group, training gains were larger at the beginning of training as compared to the end of training.

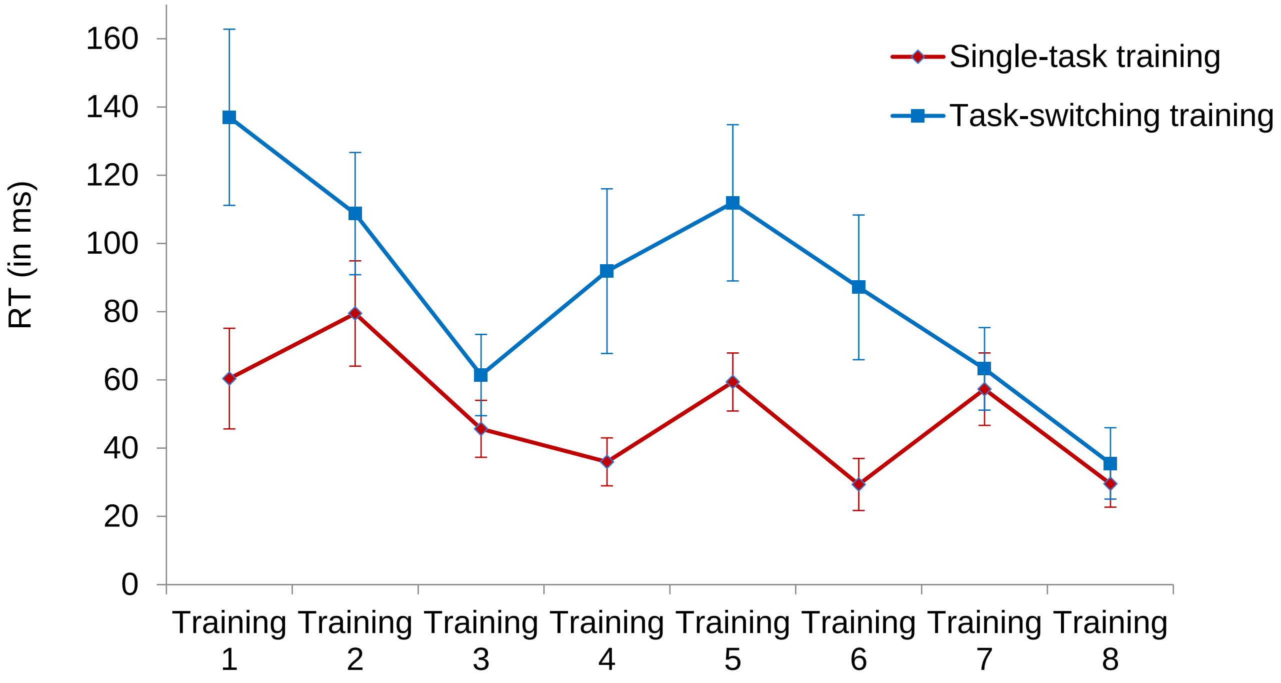

Mean RTs for Bin 1 and Bin 4 separately for repeat and switch trials in each of the eight training sessions for the task-switching group are shown in Figure 2. Due to differences in task difficulty, switching costs varied across sessions.
